# Persistent postoperative pain and healthcare costs associated with instrumented and non-instrumented spinal surgery: a case-control study

**DOI:** 10.1186/s13018-020-01633-6

**Published:** 2020-04-01

**Authors:** Sharada Weir, Mihail Samnaliev, Tzu-Chun Kuo, Travis S. Tierney, Andrea Manca, Rod S. Taylor, Julie Bruce, Sam Eldabe, David Cumming

**Affiliations:** 1 PHMR, Ltd., Bldg D, Berkeley Works, Berkley Grove, London, NW1 8XY UK; 2 Harvard Medical School, Boston Children’s Hospital, 300 Longwood Ave, Boston, MA 02115 USA; 3 Centre for Neuroinflammation and Neurodegeneration, Division of Brain Sciences, Department of Medicine, Imperial College, Du Cane Road, London, W120NN UK; 4 Centre for Health Economics, Alcuin A, University of York, York, YO10 5DD UK; 5 Institute of Health Research, University of Exeter Medical School, South Cloisters, St Luke’s Campus, Heavitree Road, Exeter, EX1 2LU UK; 6 Warwick Clinical Trials Unit, Division of Health Sciences, Warwick Medical School, University of Warwick, Coventry, CV4 7AL UK; 7 Department of Pain Medicine, Cheriton House, The James Cook University Hospital, Marton Road, Middlesbrough, BW TS4 3 UK; 8 Trauma and Orthopaedics Department, Ipswich Hospital, Heath Road, Ipswich, Suffolk, IP4 5PD UK

## Abstract

**Purpose:**

To compare rates of persistent postoperative pain (PPP) after lumbar spine surgery—commonly known as Failed Back Surgery Syndrome—and healthcare costs for instrumented lumbar spinal fusion versus decompression/discectomy.

**Methods:**

The UK population-based healthcare data from the Hospital Episode Statistics (HES) database from NHS Digital and the Clinical Practice Research Datalink (CPRD) were queried to identify patients with PPP following lumbar spinal surgery. Rates of PPP were calculated by type of surgery (instrumented and non-instrumented). Total healthcare costs associated with the surgery and covering the 24-month period after index hospital discharge were estimated using standard methods for classifying health care encounters into major categories of health care resource utilization (i.e., inpatient hospital stays, outpatient clinic visits, accident and emergency attendances, primary care encounters, and medications prescribed in primary care) and applying the appropriate unit costs (expressed in 2013 GBP).

**Results:**

Increasing the complexity of surgery with instrumentation was not associated with an increased rate of PPP. However, 2-year healthcare costs following discharge after surgery are significantly higher among patients who underwent instrumented surgery compared with decompression/discectomy.

**Conclusions:**

Although there is a not insubstantial risk of ongoing pain following spine surgery, with 1-in-5 patients experiencing PPP within 2 years of surgery, the underlying indications for surgical modality and related choice of surgical procedure do not, by itself, appear to be a driving factor.

## Introduction

The rate of spinal surgery in the UK has risen dramatically over recent years [1]. As technology has developed, so has the ability of surgeons to address more complex spinal conditions. A large proportion of patients now undergo instrumentation of their spine as part of their primary procedure. Introducing instrumentation leads to more extensive spine exposure, more soft tissue damage, potential increased infection rates, longer operative time, and increased risk of complications [2]. The rate of perioperative and postoperative complications of varying severity has been reported as high as 54%, with a screw misplacement rate of 6.5% [3]. As a consequence of the increased intraoperative complexity, it is often assumed that the rate of persistent postoperative pain (PPP), and associated health care costs, will be higher in this group compared to those undergoing non-instrumented spinal surgery. Prior research has highlighted a low, but clinically relevant, increased complication rate after lumbar spinal surgery with the introduction of pedicle screws [4], but no previous studies have compared the rates of PPP in patients undergoing instrumented vs non-instrumented spinal surgery.

We aim to address the following research questions: Are instrumented lumbar spinal fusion patients more likely to experience PPP up to 2 years following surgery compared to those undergoing non-instrumented lumbar spine surgery? Are healthcare costs higher in patients who undergo instrumented surgery compared to non-instrumented surgery, in the 2 years following surgery? The first question concerns one of the key contemporary controversies in lumbar spine surgery. This study investigating rates of PPP and associated healthcare costs will provide useful data to inform policy and practice in the UK.

## Methods

### Setting and data sources

This study employed a retrospective cohort design using the UK Hospital Episode Statistics (HES) database from NHS Digital and the UK Clinical Practice Research Datalink (CPRD). Approval was granted by the Independent Scientific Advisory Committee (ISAC) for Medicines and Healthcare products Regulatory Agency (ISAC Protocol 14-180R). These data and the study methodology were described in detail previously [1, 5].

### Study participants

Patients aged 18 and older, who underwent one or more lumbar procedures from April 2007 to March 2012 were eligible for inclusion. Index operative procedures included any single procedure or combination of discectomy/microdiscectomy, excision of lumbar intervertebral disc, laminectomy, foraminotomy, lumbar decompression (or fenestration), or instrumented lumbar fusion (including all anterior and posterior approaches as well as combined approaches). For the purposes of cleanly comparing instrumented and non-instrumented surgery, we excluded patients who underwent non-instrumented or undetermined spinal fusion. Patients were required to have a minimum 6 months pre-index surgery data without evidence of prior spinal surgery and at least 2 years of postoperative follow-up data.

### Definition of persistent postoperative pain

We categorized each surgery as a “success” (i.e., no evidence of PPP) or “failure” (i.e., evidence of PPP) depending on indications in the CPRD-HES data that the patient experienced ongoing pain, continuing past the “expected” period for recovery following index lumbar surgery. The definition of 6 months was applied for the expected postoperative recovery time. The terms “success” and “failure” are used here in the sense of success or failure of the surgery to resolve or relieve pain, rather than anatomical success or failure of the surgical procedure. As some patients may initially improve following surgery before pain returns [6–8], we chose a period of 18 months (6–24 months postoperatively) to screen for evidence of ongoing or recurrent pain. Since there are no specific diagnosis codes that may be used to identify PPP, our estimates were based upon records of additional surgery or other interventions and attendance at pain clinics. The data screening criteria used to infer ongoing or recurrent pain are summarized in Table 1. Prescription of analgesics was not included in these criteria as patients may be prescribed analgesics for other non-spinal painful conditions.

**Table 1:** Criteria for evidence of persistent postoperative pain after index lumbar surgery

Any one or more of the following	
HES inpatient	Any lumbar surgery: OPCS code 6–24 months post index surgery
HES inpatient/HES outpatient/CPRD Gold	Any surgical intervention for lumbar pain (e.g., neuromodulation, implantation of drug infusion delivery system): OPCS code or READ code at any time post index surgery
HES outpatient/CPRD Gold	At least one pain-related GP visit or specialist pain clinic visit in each of two consecutive quarters occurring 6–24 months post index surgery

### Healthcare costs

All costs were estimated from the perspective of the UK National Health Service (NHS). We first estimated the index surgery costs, including all costs incurred for the entire index inpatient episode of care. We then estimated total healthcare costs over the 24 month period after index hospital discharge by classifying health care encounters into major categories of health care resource utilization (i.e., inpatient hospital stays, outpatient clinic visits, accident and emergency attendances, primary care encounters, and medications prescribed in primary care). Respective unit costs were applied [9–12]. To account for inflation and variations in pricing over time, 2013 unit costs were applied to all years.

### Statistical analyses

To estimate rates of PPP, we computed the number of patients who met the criteria for PPP as a percentage of all patients who underwent lumbar surgery (instrumented fusion versus decompression/discectomy) within the time frame.

We compared PPP rates, and 2-year postoperative costs, of patients who underwent instrumented fusion with those who had decompression or discectomy using a control group selected with 1:1 exact matching based upon patient’s age at surgery and sex to account for any differences in likelihood of receiving instrumented surgery by age or sex. Matching was not undertaken on pre-surgical comorbid conditions. We examined the set of conditions that comprise the Charlson Comorbidity Index (CCI) evaluated in the 1-year period prior to surgery, but these were not found to be predictive of the choice of surgical mode.

Confidence intervals (95% CIs) for healthcare costs were estimated using bootstrapping to allow for non-normality of the means. The difference in PPP between cases and controls was assessed using a chi-square test of proportions.

Finally, sensitivity analyses were conducted in which the key outcome variables, PPP and costs, were adjusted to account for pre-index surgery comorbid conditions (from the CCI) using logistic regression (for the probability of developing PPP) and a generalized linear regression model (using a log link and gamma distribution, for cost estimation).

All data manipulation and analyses were conducted using SAS software, version 9.4 for Windows [SAS Institute, Cary NC].

## Results

A total of 4697 patients in the linked CPRD-HES data underwent index lumbar surgery during the UK fiscal years 2008–2012. The majority (4377 or 93.2%) of patients had decompression/discectomy, while the remainder (320 or 6.8%) had instrumented fusion. Patients who had instrumented surgery were younger (51.8 years vs. 54.9 years; *p* < 0.01) and more likely to be female (57.8% vs. 49.8%; *p* < 0.01) than those who underwent non-instrumented surgery. The age/sex-matched control group (*n* = 320) had a mean age of 51.8 years old and were 57.8% female (Table 2).

**Table 2:** Characteristics of instrumented spine patients (cases) versus others (controls) before and after matching

	Full sample			Matched case-controls		
	Non-instrumented	Instrumented		Non-instrumented	Instrumented	
	*N* = 4377	*N* = 320	*p* value	*N* = 320	*N* = 320	*p* value
Age, years	54.9	51.8	.001	52.1	51.8	.90
Gender, %	49.8	57.8	.006	62.8	57.8	.20
Myocardial infarction, %	2.6	0.9	.07	1.25	0.9	.70
Congestive heart failure, %	0.9	0.9	.96	1.6	0.9	.48
Peripheral vascular disease, %	2.0	2.2	.81	0.9	2.2	.20
Cerebrovascular disease, %	1.7	1.9	.85	1.6	1.9	.76
Dementia, %	0.1	0.3	.43	0.3	0.3	1.0
Chronic pulmonary disease, %	12.3	13.1	.66	11.9	13.1	.63
Connective tissue disease-rheumatic disease, %	2.8	4.7	.05	2.8	4.7	.21
Peptic ulcer disease, %	1.4	2.5	.13	1.6	2.5	.40
Mild liver disease, %	0.6	0.0	.16	0.0	0.0	1.0
Diabetes without complications	7.4	6.2	.43	4.4	6.2	.29
Diabetes with complications, %	0.6	0.6	.98	.6	0.6	1.0
Paraplegia and hemiplegia, %	3.2	1.6	.11	1.6	1.6	1.0
Renal disease, %	1.3	0.6	.29	0.0	0.6	.16
Cancer, %	4.0	3.4	.63	2.2	3.4	.34
Moderate or severe liver disease, %	0.02	0.0	.79	0.0	0.0	1.1
Metastatic carcinoma, %	0.4	0.6	.57	0.3	0.6	.56

One-in-five patients undergoing lumbar surgery met the study criteria for PPP. Table 3 shows that there was no statistically significant difference in the likelihood of developing PPP between those receiving decompression or discectomy versus instrumented fusion over the 2-year follow-up period (odds ratio 0.88; 95% CI 0.60–1.28).

**Table 3:** PPP rates and 2-year postoperative costs, instrumented (cases) versus non-instrumented (age- and sex-matched controls)

	Non-instrumented	Instrumented	Odds ratio/cost difference [95% CI]
Number of observations	320	320	
PPP			
Number (%)	69 (21.6%)	62 (19.4%)	0.876 [0.595–1.284]
Index hospitalization cost			
Mean	£4345	£8484	£4139 [£3737–£4563]
2-year follow-up cost			
Mean	£7597	£9423	£1826 [£285–£3244]
Total cost			
Mean	£11,942	£17,907	£5695 [£4505–£7528]

Costs of the index surgery hospital stay were almost double for patients who received instrumentation compared with age- and sex-matched controls. The mean between group difference of £4139 (CI £3737–£4563) represents the costs attributable to instrumentation (Table 3). Mean medical costs in the 2-year period following the index surgery were £1826 higher (CI £285–£3,244) for instrumented fusion patients. Putting these together, total costs attributable to instrumentation in the first 2 years were £5965 (CI £4505–£7528).

Adjusting for patient case mix did not alter these findings. In a logistic regression model adjusting for preoperative comorbidities, instrumented surgery was not associated with a greater likelihood of developing PPP (odds ratio 0.92, 95% CI 0.69–1.23). The cost difference narrowed slightly but remained statistically significant in an adjusted GLM model.

## Discussion

We reviewed 4697 lumbar surgery cases in the CPRD-HES linked databases during fiscal years 2008–2012. Of the 320 who underwent instrumented fusion, 19.4% developed PPP, an incidence similar to that of patients who had non-instrumented surgery of the lumbar spine.

Although this analysis of the UK routine data found that the surgical instrumentation of the lumbar spine does not appear to be associated with an increased rate of persistent postoperative pain up to 2 years from index surgery, the ongoing postoperative healthcare costs are slightly higher for those undergoing instrumentation when compared to age/sex-matched controls who had decompression or discectomy (excluding index surgery costs). Taking into account the index hospitalization, healthcare costs for patients having instrumented fusion cost on average almost £6000 more compared with non-instrumented procedures over the first two postoperative years.

Surgeons are aware that introducing pedicle screws involves a more prolonged operative procedure with an increased risk to the patient. The complication rate is low, however, with a 1:1000 rate of a symptomatic misplaced pedicle screw. Other complications such as infection rates also remain low at between 2 and 6%. A recent article looking at minimally invasive surgery found a rate of 0.74% [13]. The rate of rod or screw breakage is now also extremely rare. Moreover, the present analysis demonstrates that although the patient selection and the surgery itself are usually more complex, the risk of PPP may not specifically be related to the procedure. It has been previously suggested that the postoperative outcome is more related to the patient selection [14]. This is supported in previous literature which clearly demonstrates that a number of medical co-morbidities and sociodemographic factors affect postoperative outcome. This may include factors such as chronicity of the underlying or predisposing condition, other health issues, psychological factors, and employment status [15].

Our findings suggest that while there is a not insubstantial risk of ongoing pain following spine surgery, with 1-in-5 patients experiencing PPP within 2 years of surgery, the choice of surgical procedure does not, by itself, appear to be a driving factor. Further research is needed to understand what is driving the higher postoperative costs for instrumented fusion patients.

### Study limitations

There are some limitations to using healthcare records data to study risk factors and outcomes of lumbar surgery. Since there are no specific diagnosis codes that may be used to identify PPP, and our data do not contain information on pain scores used in clinical practice or postoperative imaging, it is possible that some patients were misclassified as having PPP. At the same time, we may have missed some cases of PPP since we did not consider continued use of analgesics alone to be sufficient to identify PPP.

Over the time period, spinal surgery has changed significantly. There has been an increased move towards minimally invasive surgery and an improvement in technology. This has not been considered or examined in this paper.

Our findings may be limited by the small number of patients who underwent instrumented spinal surgery during the study period in the linked CPRD-HES databases. The size of the cohort was sufficient for descriptive analyses of PPP and costs. However, future studies covering larger cohorts or longer periods of time may be useful to confirm that our findings are generalizable.

## Conclusion

We did not find that the surgical instrumentation of the lumbar spine, or the related underlying indications for this surgical modality, was associated with an increased rate of persistent postoperative pain up to 2 years from index surgery. However, the cost of the index surgery was substantively higher, and ongoing postoperative healthcare costs were slightly higher, for those undergoing instrumentation compared with similar patients who had decompression or discectomy.

## Data Availability

No additional data are available.

## Abbreviations

CCI: Charlson Comorbidity Index
CPRD: Clinical Practice Research Datalink
CI: Confidence interval
GBP: Great Britain pounds
HES: Hospital Episode Statistics
ISAC: Independent Scientific Advisory Committee
NHS: National Health Service
PPP: Persistent postoperative pain
UK: United Kingdom
